# What Constitutes Just Criticism?

**Published:** 1899-04

**Authors:** 


### Editorial.

### WHAT CONSTITUTES JUST CRITICISM?

THE active participator in the world's labor, whether he acts in the capacity of an editor of a daily paper, a journal, a reviewer of books, or as an individual, is called upon frequently to question the work of others, and this may or may not constitute criticism. Whether this be founded upon justice must be left to the judgment of those who read or listen.

Criticism, to have any value, must be founded on truth. Critics who make themselves prominent, whether as political speakers, ministers of religion, who use the pulpit for this purpose, editors, whose chief duty lies in this direction, or reviewers of books, all need to regulate their work to a high ethical standard if the critique is to make a satisfactory impression upon the judicial mind.

The political speaker who harangues his audience with exaggerated statements regarding his opponents cannot hope to find credence among intelligent auditors, no matter what their predilections may be. The minister in the pulpit who can find no better use for his eloquence than to declaim unjustly against a professor of an opposite faith loses caste among his parishioners, for charity and justice, always present in the human mind, will, in spite of bigotry and intolerance, refuse to believe all men have evil intent save those of our own faith. The editor of the daily paper, above all other men, must view both sides of every question, and dare not attach motives that may have no foundation in truth. In no other walk in life is the broad-minded man, one above the petty fault-finding spirit, more essential than here, and while this character is not always a marked factor in the press of the country, it is to its credit that the editorial instinct is rarely at fault when political prejudice is cast aside and criticism is based on the merits or demerits of the subject in hand.

It is, perhaps, more often found that the literary reviewer is the chief offender in unjust criticism. The writers of books have very good reason to fear the pen-and-ink libels upon good work. While there are honest, painstaking critics who will read books from first page to the last and then give these their best thought, it remains true that many able workers have had their hopes blasted and a bitter poison injected into their lives through crude examinations and worthless opinions of so-called reviewers. This applies with equal force to scientific books as it does to general literary work. The readers of dental periodicals must have been frequently impressed with this in review of dental books. It is common to find a book notice that gives the impression that the writer read the title-page and preface and then began to pen a review. Another will glance at these appropriate introductions to a book, and then pick here and there for a morsel to give an acrid flavor to his production. Another will see nothing in the book but good things, because it has a prominent name on the title-page. The true critic will, however, sit down to read laboriously, page by page, select the good and note those things that do not accord with his judgment, and, having thus honestly and fairly absorbed all in the volume, he is prepared to take up the pen and do justice to himself and, possibly, the author.

The individual who assumes the duty of a critic may be good, bad, or very bad in his censoriousness. There is no limit to this sort of criticism. It is the gossip of the club, social life, or of the family, and there is no remedy for it but in the cultivation of the higher altruistic ideal, a charity that covereth a multitude of transgressions.

The foregoing reflections have had their origin in many observed delinquencies in every walk of life, and have been intensified since our February issue in various ways. In that number it was deemed necessary to call attention to an article from a correspondent of the *Dental Review* who signed himself "Jesse," in which, under this anonymous signature, he sought to criticise the work of the National Association of Faculties. The reply of the editor of this journal has been criticised, in the present number, upon another page. It is not considered necessary to magnify this matter into unusual importance, and it would have been considered settled if our correspondent had not regarded it as his duty to take up the defence of "Jesse." His letter is written in such a fair spirit that it impels an answer. It is a good type of an honest criticism, although he seems to have missed the chief contention of the editorial in question. The editor of this journal has no reason to modify anything stated at that time. The article of "Jesse" belonged to a type of criticisms that makes much of little and distorts matters seemingly to effect an object. The personality of "Jesse" was then, and is now, unknown to us, and therefore, personal reflections cannot be charged. He may be a teacher of high reputation; if so, it makes the matter all the more serious to publish this statement: "As things are now, when a college is admitted to membership,—and almost any new 'old thing' can be so admitted,—it at once becomes the peer of the best."

Honest criticism is to be encouraged; indeed, it is necessary to the progress of peoples and organizations. It will be a sad day when the leading dental associations, colleges, and universities have no one to weigh the good and the evil in the balance of critical thought. The National Association of Dental Faculties is an open body. While it excludes all but members of Faculties from its deliberations, its work is open to the favorable or unfavorable opinions of all in the dental profession. It tacitly invites critical judgment of its proceedings, but resents the kind of criticism indulged in by "Jesse" as an attempt, apparently, to make things appear to the outside world as they do not to the members of that body. If he, or our correspondent, has anything that can be substantiated concerning the Association of Faculties, our pages are open, but they are not free to unsupported charges upon that body or any other that aims to do good work. All organizations are human in their origin, and consequently defective to that degree, but the motives that control to action and the reasons for their existence must be the influence that guides the pen in criticising their work.

It must be repeated that the writer of this is fully aware that dental colleges, as a whole, do not measure up to the highest standard, but this is true of all educational institutions. Not one in this country, or any other, it is assumed, comes up to the ideals of those engaged in them. It is possibly more true of dental colleges than of others, for the very excellent reason that these schools have had to be built upon the experience of the past fifty years. There were no precedents to follow save that afforded by the medical colleges, and these were often blind leaders and have led, more than once, dental teachers astray and into paths not adapted to our work.

Our correspondent seems to think that the commercial side of dental college work is peculiarly worthy of criticism. In the opinion of some others, this, if it means a business ability to make ends meet, is especially worthy of cultivation. It is folly to suppose that colleges can be maintained without endowment or some means of support. The universities are all struggling, unless State aid is afforded, to keep away from a yearly deficit. The dental schools having no endowments to depend upon must look to the returns from students to meet expenses. That some may be guilty of overstepping the bounds of legitimate work in securing students is possible, but, if so, it must be rare, as stated in a previous article, and almost certain of discovery.

The honest mind in the dental profession cannot look upon these repeated attacks upon colleges with anything but a feeling of contempt. If made by one of the dental faculty, it carries with it additional weight, and is received gladly by a certain character of mind, ever ready to see the bad, but never prepared to welcome the good in human effort. The desire of earnest thinkers is to build up to higher things. This may be by imperfect methods, but they are ever advancing and growing nearer to that perfection which is the ideal of the true-hearted in all the avocations of life.

